# Cardiovascular Aging

**DOI:** 10.31083/RCM27437

**Published:** 2025-07-23

**Authors:** Marco Tana, Rachele Piccinini, Livia Moffa, Ettore Porreca, Fernando Tana, Claudio Tana

**Affiliations:** ^1^Internal Medicine and Cardiovascular Ultrasound Unit, Medical Department, SS. Annunziata Hospital, 66100 Chieti, Italy; ^2^School of Internal Medicine, G. D’Annunzio University, 66100 Chieti, Italy; ^3^Oncology Unit, School of Medical Oncology, Polytechnic University of the Marche, 60121 Ancona, Italy; ^4^Infectious Diseases Unit, School of Infectious Diseases, G. D’Annunzio University, 66100 Chieti, Italy; ^5^AUSL Pescara, 65125 Pescara, Italy; ^6^Geriatric Clinic, SS Annunziata Hospital, 66100 Chieti, Italy

**Keywords:** cardiovascular aging, cardiac amyloidosis, heart failure, ejection fraction, oxidative stress

## Abstract

Aging is a slow, progressive, and inevitable process that affects multiple organs and tissues, including the cardiovascular system. The most frequent cardiac and vascular alterations that are observed in older adults (especially patients aged ≥80 years) are diastolic and systolic dysfunction, progressive stiffening of the vascular wall and endothelial impairment usually driven by an excess of extracellular matrix (ECM) and profibrotic substances, reduced levels of matrix metalloproteinases (MMPs), or by amyloid and calcium deposits in myocardium and valves (especially in aortic valves). Moreover, deformation of the heart structure and shape, or increased adipose tissue and muscle atrophy, or altered ion homeostasis, chronotropic disability, reduced heart rate, and impaired atrial sinus node (SN) activity are other common findings. Interestingly, aging is often associated with oxidative stress, alterations in the mitochondrial structure and function, and a low-grade proinflammatory state, characterized by high concentrations of cytokines and inflammatory cells, without evidence of infectious pathogens, in a condition known as ‘inflammaging’. Aging is a well-recognized independent risk factor for cardiovascular disease and easily leads to high mortality, morbidity, and reduced quality of life. Recently, several efforts have been made to mitigate and delay these alterations, aiming to maintain overall health and longevity. The primary purpose of this review was to provide an accurate description of the underlying mechanisms while also exploring new therapeutic proposals for oxidative stress and inflammaging. Moreover, combining serum biomarkers with appropriate imaging tests can be an effective strategy to stratify and direct the most suitable treatment.

## 1. Introduction

Cardiovascular aging is a progressive and inevitable biological process that profoundly affects both the structure and function of the heart and vascular system. As the global population ages, age-related cardiovascular conditions—such as heart failure, aortic stenosis, and vascular stiffening—are becoming increasingly prevalent and represent a major burden on healthcare systems worldwide.

This review article explores the complex pathophysiological mechanisms underlying cardiovascular aging, including myocardial remodeling, extracellular matrix (ECM) accumulation, altered matrix metalloproteinase (MMP) activity, endothelial dysfunction, and vascular wall stiffening. A central focus is placed on the role of oxidative stress and chronic low-grade inflammation—commonly referred to as “inflammaging”—as key drivers of these degenerative processes. These molecular pathways contribute to reduced myocardial contractility, impaired vascular compliance, and increased susceptibility to atherosclerosis and arrhythmias.

We also highlight the impact of mitochondrial dysfunction, cellular senescence, and maladaptive neurohormonal activation on cardiovascular health in older adults. Special attention is given to emerging diagnostic tools, including circulating and urinary biomarkers, which may allow for early identification and risk stratification of patients with age-related cardiovascular changes. The potential clinical relevance of molecules such as B-type natriuretic peptide (BNP), interleukin (IL)-6, high-sensitivity cardiac troponin T (hs-cTnT), and fibroblast growth factor 21 (FGF21) is discussed, alongside non-invasive imaging techniques. In addition to pathophysiological insights, the review outlines a range of preventive and therapeutic strategies aimed at delaying or mitigating cardiovascular aging. These include lifestyle modifications—such as regular physical exercise and dietary interventions—as well as pharmacological approaches targeting inflammation, oxidative stress, and MMP regulation. Ultimately, the review provides a thorough and up-to-date synthesis of current knowledge in the field of cardiovascular aging, with the goal of informing future research and improving care for an increasingly elderly population.

## 2. Age-Related Changes

Aging is a slow, progressive, and inevitable process that involves several organs and tissues, including the cardiovascular system, and is commonly considered an independent risk factor for cardiac and vascular diseases [1, 2, 3].

By 2030, an increase in population growth and ageing (approximately 20% of the entire population will be aged 65 years or older) will promote additional rises in cardiovascular diseases (CVDs) of at least 40% in all deaths. Thus, this scenario is associated with increased therapeutic costs and a negative economic impact on hospitalizations and the entire health system [4].

A constant and gradual decline in various physiological processes is a natural result of aging, and it is generally defined as a complex process that begins in the fourth decade of life, resulting from a combination of social, biological, and psychological factors [5].

Alterations in metabolism and organ and tissue functions can be used to characterize human aging. In older adults, the size of the trachea, bronchi, and number of alveoli are reduced, along with a decrease in vital capacity (VC) and total pulmonary capacity (CPT), and lung compliance. These alterations easily lead to an increase in dead space and reduced blood oxygenation. Moreover, the function and motility of respiratory cilia are impaired, thus resulting in higher susceptibility to infections [6].

The gastrointestinal system is affected by aging, with reduced gut and stomach motility, a reduction in teeth, saliva, peristalsis, and pancreatic function, as well as dysfunctional regeneration activity by the liver.

A decrease in strength and muscle mass, as well as bone density, leads to higher susceptibility to osteoporosis and fractures, which are common changes in the musculoskeletal system [7]. Moreover, the genitourinary system is easily impaired through reduced kidney filtration function and loss of sphincter activity [8]. Meanwhile, the tegumentary system and sensory organs are involved in alterations such as skin atrophy, dryness of the mucous membranes, reduced vision, and reduced hearing ability. The ability of the skin to respond to damage or inflammation is reduced, as are nerve fibers and sweat glands [9]. In addition, psychosocial maladaptation to the environment, with a sense of loneliness, a hostile attitude, and the need to depend on a caregiver, are all significant consequences of aging. Furthermore, as it is well known, aging involves various alterations in the nervous system ranging from neuronal atrophy or cell death, reduced memory, increased white matter [10], which can lead to the onset of neurodegenerative diseases, such as Alzheimer’s disease (AD), Parkinson’s disease (PD), Huntington’s disease (HD), and frontotemporal lobar dementia (FLTD) [11].

## 3. Age-Related Changes in the Cardiovascular System

### 3.1 Role of MMPs and ECM

The most frequent cardiac and vascular alterations observed in older individuals (especially patients aged ≥80 years) are diastolic and systolic dysfunction of the heart, progressive stiffening of the vascular wall, and endothelial dysfunction [12, 13].

Several authors have described how various cardiac and vascular diseases are linked to an altered balance between MMPs, ECM, and tissue inhibitors of metalloproteinases (TIMPs) [14, 15].

MMPs belong to a large family of zinc-dependent endopeptidases responsible for demolishing protein substrates of the ECM, such as collagen and elastin. They are commonly made up of a catalytic sequence, a hinge region, and a hemopexin sequence and are classified according to their substrates (gelatinase, collagenase, etc.). Commonly, these proteinases are secreted in an inactive form by vascular smooth muscle cells, fibroblasts, and leukocytes [16].

A disequilibrium between MMPs and TIMPs has been described by various authors in patients with arterial hypertension, in the formation of arterial plaques, in the remodeling of varicose veins and aortic aneurysms [17] and some exogen MMP inhibitors (MMPi) showed promising results in the management of vascular diseases [17, 18, 19].

Specifically, MMPs play a key role in arterial remodeling through various mechanisms, including endothelial inflammation, degradation of elastin fibers, arterial wall calcification and fibrosis, as well as alterations in the adventitial wall. Endothelial inflammation appears to be associated with cell necrosis and apoptosis (resulting from p53 and caspase activation), thrombosis, platelet aggregation, reduced nitric oxide production (involving heat shock protein 90 catabolism), and alterations in vasodilation. Moreover, MMPs can directly destroy elastin fibers through the phosphorylation of proteins involved in elastogenesis, such as ERK-1/2, or through direct digestion. In addition, calcification and fibrosis of the arterial wall are due, respectively, to both increased cakpain-1, which can physiologically reduce the activity of calcification inhibitors, and to direct activation of tissue growth factors (TGFs), such as TGF-beta1. Interestingly, the destruction of elastin fibrils leads to the release of a TGF-binding protein, thereby enhancing its activation, collagen production, and fibronectin. Even the thickening of the adventitial wall is attributed to the actions of MMPs, TGF, and inflammatory cells, resulting in a negative feedback loop [20].

The ECM can be viewed as a robust, three-dimensional macromolecular mechanical support present in all tissues and organs, composed of various interconnecting fibers that reinforce the cytoskeletal architecture. In addition to being a valid anchor structure, the ECM modifies and adapts in response to different mechanical stresses, thereby strengthening the rigidity of the structure to which it is attached. In physiological conditions, ECM is a solid protein structure composed of collagens, proteoglycans/glycosaminoglycans, fibronectin, elastin, and other glycoproteins, as well as laminin, which aligns and supports myocytes. MMPs or various other proteases are responsible for the constant remodeling of this structure and are important in preventing overproduction [21]. Conversely, some substances, such as transforming growth factor-β (TGF-β), can increase the levels of ECM and reduce MMPs [22, 23].

### 3.2 Vascular Aging

Vascular involvement in older adults can be easily summarized by two key factors: wall stiffening and endothelial dysfunction [24]. A reduction in elastic fibers, accompanied by the deposition of collagen and calcium, is a relatively common finding in the arterial vessels of older people [25].

Specifically, a high elastolytic activity is documented in the walls of older individuals and frail subjects [26]. A high activity of proteases with elastolytic properties, such as MMP-1, -2, -9, -13, and -14, or cathepsins has also been observed [20, 27, 28, 29].

Several studies have reported an imbalance between MMPs/TIMPs and/or MMPs/ECM in older individuals, and among these alterations, MMP-2 has been shown to be strictly related to cardiovascular disease [26, 27].

Interestingly, these peptidases are, in turn, upregulated by oxidative stress, growth factors, or inflammatory stimuli [30]. A close connection between elastin degradation products and the activation of wall repair mechanisms has also been described. Amorphous elastin degradation products can act as an inflammatory chemotactic stimulus for cells, such as leukocytes, smooth muscle cells, and fibroblasts, or even exhibit angiogenic activity. Thus, continuous exposure to elastases during aging can lead to a chronic inflammatory state, known as ‘inflammaging’, and, again, a negative feedback loop [31].

The progressive reduction in wall elasticity and increased stiffening lead to elevated blood pressure, morbidity, and mortality [32, 33]. Meanwhile, other alterations have also been described, including reduced sensitivity to vasoconstrictors and vasodilators, as well as a marked reduction in neoangiogenesis. As mentioned above, the following endothelial dysfunctions have also been studied: impaired wall integrity and abnormal responses to damage, leading to a greater susceptibility to atherogenesis [34]. Coronary arteries are also involved in these alterations.

### 3.3 The Aging Heart

In older adults, myocytes are constantly solicited by oxidative stress, resulting in reduced contractile tissue, an increase in fibroblast cells, compensatory hypertrophy of remaining tissue, decreased elasticity, and diastolic dysfunction [1, 35, 36]. The response of cardiomyocytes to apoptosis is also reduced, resulting in impaired cell renewal [37].

Structural and functional alterations of the ECM are commonly found in the hearts of older adults. Moreover, any alteration of the levels of ECM, MMPs, and profibrotic substances in older individuals leads to an excess of ECM and stiffness of vessel walls, myocardium, and reduced contractility [22, 38, 39]. In addition, several studies have documented the importance of deformation of heart structure and shape (ventricular septum), or amyloid and calcium deposits in myocardium and valves (especially the aortic valve), and muscle atrophy with increased adipose tissue [40].

A certain degree of chronotropic disability, characterized by reduced heart rate and atrial sinus node (SN) malfunction, especially in individuals with coexisting diabetes [41], is a common finding in older adults. This is primarily related to the loss of cells, accompanied by fibrotic alterations in the sinoatrial (SA) node and conduction elements [42]. These elements cause common conditions, such as arrhythmias or a diminished autonomic drive [43].

Other functional disorders have been recognized, including reduced myocardial utilization of calcium (Ca^2+^), a reduction in the endothelial nitric oxide (NO) concentration, and dysfunction of adrenergic capacity with reduced sympathetic tone at both cardiac and vascular sites [1, 44].

Moreover, the aging heart can be indirectly affected by the vascular system: a ventricular afterload and decreased output are relatively common findings [45] as a direct consequence of pulse pressure widening, arterial wall stiffness, and systolic arterial hypertension [46]. The most common pathways involved are hyperglycemia and insulin resistance, collagen damage by oxidative stress, and fibrogenesis resulting from alterations in the renin–angiotensin–aldosterone system (RAAS) [47, 48].

## 4. Cellular Aging, Inflammatory Responses, and Oxidative Stress

**Fig. 1.**
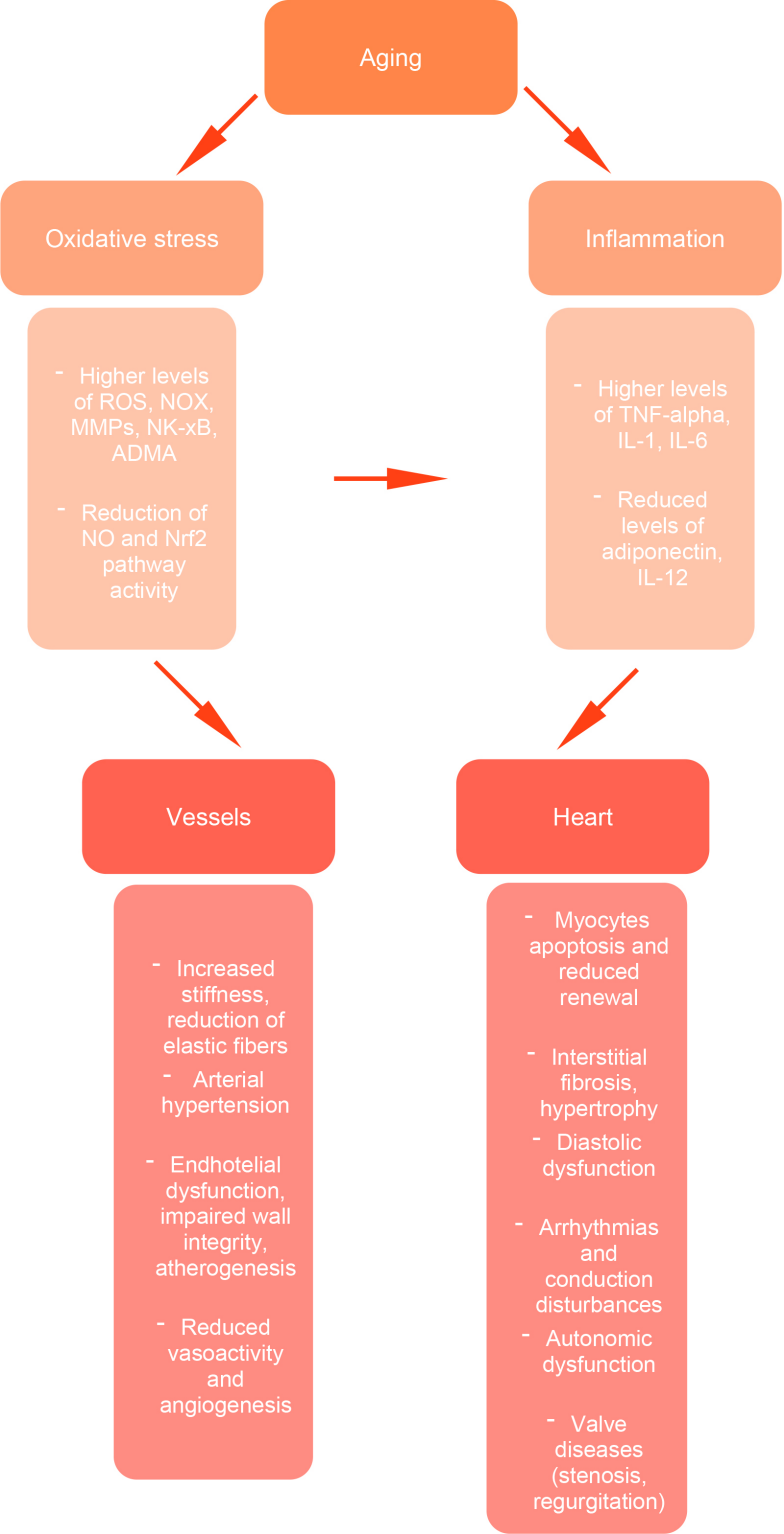
**Cardiovascular aging: pathogenetic mechanisms**. Aging promotes oxidative stress and chronic inflammation, leading to increased levels of reactive oxygen species (ROS), matrix metalloproteinases (MMPs), and proinflammatory cytokines (TNF-α, IL-1, IL-6), along with a reduction in protective pathways, such as nitric oxide (NO) signaling and Nrf2 activity. These alterations contribute to structural and functional damage at both the vascular level (increased stiffness, endothelial dysfunction, impaired angiogenesis) and the cardiac level (myocyte apoptosis, interstitial fibrosis, diastolic dysfunction, arrhythmias, autonomic dysregulation, and valve diseases). Together, these processes underlie the age-related decline in cardiovascular function. NOX, NADPH oxidase; MMPs, matrix metalloproteinases; ADMA, asymmetric dimethylarginine; IL, interleukin.

Further, cellular senescence and aging are frequently associated with a low-grade pro-inflammatory state in the absence of evident infectious pathogens, called ‘inflammaging’, which is characterized by high concentrations of TNF-α, macrophages, monocytes, and lymphocytes [49, 50, 51, 52, 53]. Fig. 1 shows the complex interplay between oxidative stress, chronic inflammation, and their downstream effects on the vascular and cardiac systems, highlighting key molecular pathways and structural changes that drive age-related cardiovascular dysfunction.

The etiology and physiopathological mechanisms remain unknown. According to the antagonistic pleiotropy theory of aging, an inflammatory state in adolescence and adulthood could have positive and beneficial effects, because it would have a contrasting action against pathogens; however, this inflammatory state can then also cause damage if it is perpetrated chronically in older adults [50, 51], being associated strongly with a worse prognosis [54], high mortality and morbidity [51], and includes cardiac alterations, such as myocardial hypertrophy, interstitial fibrosis and diastolic dysfunction [55]. Mitochondria are the primary structures that modulate oxidative stress, and therefore, cellular aging is indirectly affected. Meanwhile, mitochondria normally respond through various adaptive control mechanisms that are activated in response to perceived oxidative stress. These include the unfolded protein response specific to mitochondria (UPRmt), the formation of mitochondrial-derived vesicles (MDVs), and mitophagy, which removes damaged or dysfunctional mitochondrial components or entire mitochondria. In cases of irreversible damage, cells may also activate programmed cell death pathways, such as apoptosis or necrosis [2].

Under normal conditions, oxidative stress and reactive oxygen species (ROS) usually enhance the activation of the antioxidant response, which includes the activation of signaling pathways such as NF-κB, the mitogen-activated protein kinase (MAPK), and Keap1–Nrf2–antioxidant pathway, a nuclear factor whose activation normally induces transcription of antioxidant genes [48]. All these mechanisms trigger a series of beneficial adaptive responses, such as cell growth, autophagy, and inflammation.

However, increased oxidative stress and an impaired cellular response to ROS are commonly observed in older adults. This increase is associated with elevated serum levels of ROS, resulting in DNA damage, alterations in various mitochondrial proteins, and further amplification of ROS production, establishing a self-perpetuating vicious cycle [1].

In detail, the most common alterations are reduced mitochondrial function and high levels of NADPH oxidase (NOX); both of which activate an increase in ROS, MMPs, and NK-xBA (ADMA), and a reduction in NO and the Nrf2 pathway [48].

Additionally, protein carbonylation and relative formation of ketones and aldehydes are other irreversible consequences of oxidative stress in older adults [56]. Interestingly, high levels of carbonylated proteins are found in patients with PD [57], AD [58], cardiac amyloidosis (CA), especially the transthyretin form, thus suggesting a strong link between amyloid fibrils, oxidative stress, and age-related disease [59, 60, 61].

Post-transcriptional changes in chaperones and proteins can damage transthyretin, thus resulting in their extracellular deposition, such as in the myocardium, with a direct toxic activity in a vicious circle [62, 63].

In older people, mitochondria appear swollen with few mitochondrial crests and are characterized by impaired ATP metabolism and accumulation of intralysosomal lipofuscin. Moreover, altered and swollen mitochondria tend to become larger and less capable of performing phagocytosis. Similar alterations also occur in smooth muscle cells with additional anatomical and functional repercussions on the arterial vascular walls [62, 63, 64].

Interestingly, the activity of peroxisome proliferator-activated receptor gamma coactivator 1α (PGC-1α), which is involved in mitochondrial homeostasis and the equilibrium between biogenesis and degradation, appears to be reduced in older adults, resulting in impaired heart function and activity [65].

In addition, not only systemic and local age-related changes, but also comorbidities commonly seen in frail individuals, such as diabetes mellitus, obesity, dyslipidemia, and hypertension, can have a direct impact on the heart [66].

In a prospective study conducted by Vasan *et al*. [66] with a mean follow-up of 5 years, low serum levels of insulin-like growth factor-1 (IGF-1) were strongly related to cachexia, ventricular dysfunction, and worsening of heart failure in older subjects.

Recently, growing evidence has emerged regarding the role of microRNAs as regulators in the pathogenesis and development of CVDs, as well as their involvement in senescence pathways and comorbidities [67, 68, 69].

## 5. Potential Research Opportunities Using Novel Biomarkers for the Early Diagnosis of Cardiovascular System Aging

As previously mentioned, aging is strongly related to CVDs and is a well-recognized independent risk factor [1, 2, 3, 4, 5]. Older individuals have higher serum levels of BNP, hs-cTnT, C-reactive protein (CRP), and IL-6 compared to younger subjects [46, 47, 48, 49, 50]. Secretory molecules easily detectable in biological fluids such as urine or blood could be commonly used in clinical routine as biomarkers for the early diagnosis of cardiovascular disease and as a prevention strategy, especially in vulnerable populations, such as older individuals [70].

Higher values of serum BNP are related to a reduced left atrial (LA) reservoir and conduit strain rate, as measured by speckle-tracking echocardiography, and may correlate with heart failure in older adults [50].

Several authors have proposed urine as a simple and cost-effective biological fluid for diagnostic purposes, as it can be easily collected even in outpatient settings [70]. Interestingly, high BNP values in the urine are strongly related to CVDs [71].

As in the heart, biomarkers have also proven useful in vascular ageing. Indeed, elevated levels of circulating fibroblast growth factor 21 (cFGF21) are more frequently observed in older adults [72]. According to a systematic review and meta-analysis by Zhang *et al*. [73], these levels are strongly associated with an increased risk of CVDs.

Circulating levels of CRP, IL-6, IL-1, or oxidized low-density lipoprotein (ox-LDL) are increased in vascular ageing and are closely related to inflammaging and oxidative stress [74, 75].

In addition, several authors have found an interesting relationship between brachial–ankle pulse wave velocity (PWV), blood levels of fibulin-1, and vascular age, suggesting a possible role for this biomarker in arterial stiffness and cardiovascular burden [76, 77].

Vascular healing is also compromised in the aging process: bone marrow-derived endothelial progenitor cells (EPCs), which normally contribute to vascular wall repair and neoangiogenesis, exhibit both reduced numbers and impaired function in older adults [78, 79, 80].

## 6. Intervention Measures

Cardiovascular aging can be slowed by general preventive strategies, such as an adequate dietary regimen, regulation of caloric and sodium intake, as well as specific nutrients. In addition, both regular physical exercise and the cessation of alcohol and smoking abuse, or the reduction of psychosocial stress, can permit the delay of cardiovascular ageing [1, 2].

Chen *et al*. [81] summarized the positive effects of regular physical activity on various signaling pathways in the cardiovascular system. El Assar *et al*. [48] highlighted the benefits of inflammation (reduced levels of TNF-α, IL-6, and higher levels of IL-10 and adiponectin), on oxidative stress (decreasing levels of ROS and higher levels of Nrf2), and various effects on mitochondrial activity (especially an increase in PGC-1α activity).

In addition, a physiological hypertrophic response of myocardial tissue, which would be less vulnerable to ischemic insult and cardiac remodeling, was observed after physical exercise [81]. Moreover, constant and regular aerobic activity can have a cardioprotective effect through the regulation of RNA and various signaling cascades [81].

Physical activity upregulates the sympathetic tone and downregulates the parasympathetic tone, resulting in increased heart rate, blood flow to the heart, and improved contraction and systolic activity through the Frank–Starling mechanism [82, 83].

Specifically, moderate physical exercise has proven effective in reducing glucose and insulin levels, blood pressure, and body mass index (BMI) [84, 85], regardless of age group [86].

A reduction in myocardial oxidative stress driven by reduced ROS and higher antioxidants levels, together with augmented cardiac, neoangiogenesis and lymphangiogenesis activities derived from vascular growth factors and endothelial vasodilatation, in addition to metabolic changes, such as glucose utilization or adenosine triphosphate (ATP) production, are all positive benefits on the cardiovascular system induced by active exercise, and are also observed in the oldest population [87, 88].

In addition to general measures, other authors have emphasized the importance of exogenous agents that can modulate the activity of MMPs. Additionally, biological and endogenous inhibitors, such as TIMPs and MMPs, can be downregulated by different exogenous agents (biological or pharmacological), including statins, captopril, sulfonamides, or cilostazol [89, 90].

Thus, MMP expression and its function are regulated by numerous factors, including biological effectors, endogenous inhibitors, epigenetic regulators, miRNAs, and pharmacological agents. Recently, several authors have utilized pharmacological modulators of MMPs to prevent or modify the course of CVDs [30, 89].

## 7. Conclusions and Future Directions 

Early identification of cardiac and vascular ageing biomarkers, along with targeted treatment of inflammatory pathways and modulation of key signaling molecules, or early management of comorbidities, may offer significant benefits in older populations. These strategies could not only aid in cardiovascular prevention but also help slow the progressive and continuous deterioration of the systems involved in the ageing process.

Inflammation and oxidative stress appear to share common molecular pathways, and several promising pharmacological treatments with both antioxidant and anti-inflammatory properties may represent reasonable therapeutic options in older adults. In addition, modulation of MMP inhibitory activity could have beneficial effects in reducing and preventing CVDs, even in older populations.

Nonetheless, general lifestyle interventions, such as regular physical activity, a balanced diet, and caloric restriction, can significantly support cardiovascular health, particularly in older adults. A deeper understanding of the underlying signaling pathways may aid in identifying specific therapeutic targets in the near future; however, further studies are required to validate these approaches.
